# Priority-setting in health research in Iran: a qualitative study on barriers and facilitators

**DOI:** 10.1186/s12961-018-0313-1

**Published:** 2018-07-02

**Authors:** Abbas Badakhshan, Mohammad Arab, Arash Rashidian, Neda Mehrdad, Kazem Zendehdel

**Affiliations:** 1 Department of Health Management and Economics, School of Public Health, Tehran University of Medical Sciences (International campus), ᅟTehran, Iran; 2 Health Management and Social Development Research Center, Golestan university of Medical Sciences, Gorgan, Iran; 3 Department of Health Management and Economics, School of Public Health, Tehran University of Medical Sciences, Tehran, Iran; 4 Endocrinology and Metabolism Research Center, Endocrinology and Metabolism Clinical Sciences Institute, Tehran University of Medical Sciences, Tehran, Iran; 5 Knowledge Utilization Research Center, Tehran University of Medical Sciences, Tehran, Iran; 6 Cancer Research Center, Cancer Institute of Iran, Tehran University of Medical Sciences, Tehran, Iran; 7 Cancer Biology Research Center, Cancer Institute of Iran, Tehran University of Medical Sciences, Tehran, Iran

**Keywords:** Health research, Iran, management, priority-setting

## Abstract

**Background:**

Priority-setting is a complicated and time-consuming process; however, if appropriately conducted, it could efficiently divert resources to the most important studies. A considerable body of evidence indicates that priority-setting measures in health research taken so far in Iran have not satisfied decision-makers, policy-makers, funders, communities, or even researchers. This study was designed to explore the flaws of these measures and their deciding factors.

**Methods:**

We conducted semi-structured interviews with 23 key participants and used a thematic data-analysis approach to analyse verbatim transcripts and documents. Our interviewees, who were skilful at conducting health research and worked as managers at different levels of the health system, were selected using a purposeful sampling. We asked about their experiences of priority-setting in health and relevant challenges and asked for recommendations. These semi-structured interviews were taped, transcribed and analysed in terms of content and themes using the MAXQDA10 qualitative data-analysis software.

**Results:**

With regard to priority-setting facilitators and barriers, four themes were extracted, namely managerial factors, structural factors, motivational factors, and process factors. Managers’ commitment, consideration of intellectual property, compliance with superordinate rules, and provision of a definition of reliable criteria were among the facilitators. The rapid turnover of managers, inefficiency of criteria for faculty promotion, and disregard of appeal mechanisms were examples of the barriers.

**Conclusion:**

It is important to consider appropriate regulations and motivations to provide research priorities and divert scarce resources to them. In addition, it is necessary to improve the knowledge and skills of researchers and research administration offices on priority-setting methods, thereby enhancing priority-oriented research projects.

## Background

The health research system has four main functions, namely those of setting priorities, enhancing research capacity, defining research norms as well as standards, and translating evidence into action [1]. Priority-setting has been introduced as a discussion [2], a consensus-building process [3], and a political process [4]. It has also been defined as rationing or allocating limited resources to people or programmes in a competitive situation [5–7]. Priority-setting depends on environmental and organisational conditions. Almost all of those who have experienced priority-setting regard it as a complicated and time-consuming process, with ethical and political aspects [8, 9].

Research priority-setting is conducted for a variety of reasons, including controlling expenditures in the field of priorities and decreasing the 10/90 gap in health financing, wherein global evidence shows that 90% of health expenditures are allocated to those diseases or risk factors that cause only 10% of the global burden of disease [10–15]; promoting science, technology and innovation for different aspects of diseases or risk-factor control; creating more opportunities for international and intersectoral collaborations in order to solve health problems; taking advantage of negotiations with partners to obtain targeted funding; and diverting technical, financial and human resources into the control of diseases or risk factors. Further, various methods have been introduced for research priority-setting. Selecting what research should be done could be accomplished according to the burden of disease, yet statistics show mismatches between disease burden and research funding [16]. Some modelling techniques, such as value of information, or economic approaches like programme budgeting and marginal analysis have also been introduced, but due to scarcity of related information and the complexity of methods, especially in low- and middle-income countries, these are rarely used. Over the past decade, different approaches to targeting resources to health research in developing countries have been introduced by international health organisations and agencies. WHO and its affiliated organisations and initiatives, such as the Council on Health Research for Development (COHRED), the Special Programme for Research and Training in Tropical Diseases, and the Child Health and Nutrition Research Initiative, have recommended approaches such as essential national health research, combined approach matrix, the COHRED management approach, and the Child Health and Nutrition Research Initiative and Special Programme for Research and Training in Tropical Diseases methods. These approaches are apparently aimed to deviate traditional research agenda-settings from scientific autonomy to public engagement.

Obviously, achieving these valuable and important goals depends on the success of research priority-setting. First of all, a consensus should be reached on the meaning of a successful research priority-setting [12, 17–19]. Surprisingly, the only point on which there is complete consensus is that there is no complete consensus on a gold-standard method for research priority-setting [5, 20–22]. The abovementioned organisations, along with the Global Forum for Research and some independent experienced researchers, have attempted to define the requirements for successful research priority-setting through the introduction of methods or assessment checklists. A body of evidence indicates that research priority-setting efforts have failed to achieve their goals due to various factors, including the number of stakeholders and their type of participation, criteria set, evidence required, scope of priority-setting, levels of priorities, and ways of establishing an evaluation phase [23–28].

Developing countries, including Iran, suffer from a gap between their research expenditures and their research priorities [26]. Health research resources in Iran are scarce and, while developed countries devote approximately 2.2% of their Gross Domestic Product to health research, the share of health research in Iran is just of approximately 0.39% [29]. A study in 2007 showed that health research priorities in Iran accounted for only 15% of health research expenditures [26]. Additionally, past evidence demonstrates that health research in Iran is not in line with the identified priorities [24]. The weakness of knowledge management, insufficient knowledge creation, the lack of a consistent database of domestic health research results, inadequate annual growth rate of health researchers, the lack of communication between the producers of research findings and consumers, a mismatch between conducted research and consumers’ needs, and problems of research management are other reported defects of health research in Iran [28]. Several research priority-setting procedures have been conducted in Iran; however, they have failed to solve problems and health research projects are not usually carried out according to national or regional priorities. Therefore, it is essential to understand the barriers and facilitators affecting these issues. The present study is a qualitative study on these assessment of these factors in Iran, a developing country that has invested in health research heavily in recent years.

## Methods

We conducted semi-structured interviews with 23 key participants and collected relevant literature and documents. A thematic data-analysis approach was utilised to analyse the verbatim transcripts and documents. Participants were selected through purposeful sampling, meaning that the criteria for inclusion of informed participants were having experience in the macro, meso and micro level of research management and being engaged in health research priority-setting. A list of researchers involved in health research priority-setting was extracted from a literature review of domestic studies. Since three of the researchers, due to their previous research responsibilities, were familiar with the experience and ability of the faculty members of the universities, a list of this group was also prepared. This list merged with the another list that previously prepared based on literature review. Interviews started with those who satisfied both criteria. A snowballing technique, wherein participants were also selected based on the main participants’ recommendations, was also applied. Table 1 shows the final composition of the interviewees. Managers who engaged in this study were selected from the macro-, meso-, and micro-levels of the health system, working as healthcare or health-research managers, including current and previous Deputies to the Research and Technology in the Ministry of Health and Medical Education, Head of the WHO Representative Office in Iran, Chairman of the Iran Health Insurance Organization board of directors, Head of National Institute of Health Research, Research Deputy of National Institute for Medical Research Development, deans and deputies of medical universities, and deans and deputies of health research centres. Interviews were conducted in each participant’s office after briefing them on aims of the research, ensuring their anonymity and obtaining their informed consent. Each interview lasted approximately 1–1.5 hours. One or two researchers attended the interview sessions. Saturation was achieved after 23 interviews. The literature was reviewed to determine measures of a successful priority-setting. The measures determined were summarised and used in a semi-structured self-administered questionnaire, which was used in the interviews. This questionnaire was reviewed and corrected by five experienced researchers.

**Table 1:** The participants, characteristics

SEX		Researcher^a^				Manager^b^					
male	female	Basic	clinical	Socio- emotional	Health system	MOHME^c^	Universities			Research centers	Health sector (outside MOHME)
							Dean	Deputy	others		
15	8	2	11	5	5	5	1	3	1	9	2

^a^Epidemiologists are considered as clinical researchers

^b^Two researchers were not mangers but one of them has managerial experience at MOHME

^c^Consist of different levels: from Deputies to experts

### Data analysis

Interviews were taped, transcribed and then thematically analysed. An approach combining deductive and inductive frameworks was used to develop a thematic framework, code the transcribed text and analyse the data. During each interview, brief notes were taken regarding the atmosphere of the interview, the interviewee’s emphasis on a word or phrase, and the interviewee’s sympathy, interruptions and body language. After each session, the notes were used to prepare field notes, which were analysed simultaneously to the interviews to improve understanding and meaning. Prior to analysis, each transcribed interview was labelled with a number attributed to the related interviewee in a separate sheet to ensure confidentiality.

The data analysis consisted of a thematic analysis organised into three phases, namely open, axial and selective coding. For the thematic analysis, MAXQDA 10 was used. MAXQDA is a programme designed for analysing data, texts and multimedia related to computer-assisted qualitative and mixed methods in scientific, academic and business institutions. In open coding, the entire interview was read to get a sense of the whole interview and the initial impression. Next, it was segmented by identifying parts of the data related to an idea, e.g. Criteria and Stakeholder. The text units were selected and some descriptive notes were assigned to them. In axial coding, concepts were organised into overarching themes, such as managerial incentives, based on the participants’ emphasis on some words in responses and also their repeated use of particular words. Finally, in selective coding, descriptions of the themes were provided in the participants’ own words. We addressed the validity of our findings in three ways. First, the principal researcher worked with a second senior researcher in developing a coding framework and checking it. Second, interim results were presented to the participants to enhance reflexivity and insure presumptions, experiences and personal bias were taken into consideration. Third, all research activities were rigorously documented to permit a critical appraisal of the methods. In addition, data was also collected from different resources including transcribed interviews and field notes.

## Results

The interviews were coded consecutively, resulting in 1553 codes, which, after re-reading and considering the field notes, was reduced to 1262. As shown in Table 2, eventually, these codes were categorised into four themes and 29 sub-themes, which are expanded on in the following lines.

**Table 2:** Thematic framework: Factors explaining what influences conducting and implementing priority-setting results

Facilitator	Facilitator and barrier	Barrier
Theme 1: Managerial factors		
Meritocracy in management	Individual willingness	Rapid turnover of managers
Commitment of managers		Scarcity of knowledge about HRPS
Theme 2: structural factors		
Publicising HRPS results	Role of media	Centralised decision-making
Using an automated system		Integration of heath with medical education
		Lack of a national innovation system
		Stewardship – absence of a research map (research puzzles)
		Stewardship – inappropriate leadership of research
		Stewardship – lack of transparency in other sectors
		Stewardship – no standardisation
		Lack of evidence about research gaps
		No relation with industries
Theme 3: Motivational factors		
Considering intellectual property	Limiting research budgets to priorities	Inefficiency of faculties’ promotion criteria
		Narrow time limit for PS
Theme 4: Process factors		
Defining reliable PS criteria	PS approaches	Considering PS as a one-time activity
Alignment with high level rule		Generalisation
Stakeholders – ways of engagement		Ignoring appealing mechanisms
Stakeholders – end users		Lack of a efficacious evaluation system
Stakeholders – NGOs		Scientific autonomy
Stakeholders – funders		
Stakeholders – policy-makers		
Defining scope of PS		

*HPSR* health research priority-setting, *PS* priority-setting

### Theme 1: Managerial factors

The rapid turnover of health managers affects priority-setting activities significantly. According to a participant, “*everyone looks for great achievements in their managerial period and, since it is too short, they want to get them as soon as possible*” (P13). On the other hand, a short managerial period limits managers and they cannot get involved in complicated and time-consuming activities such as priority-setting. “*I am a manager today but might not be tomorrow. So, when I am to work in an unstable position, I have to do unexpected tasks*” (P5).

Disregarding the hierarchy of academic degrees while offering a managerial position in a scientific institution would disappoint subordinates and prevent them from getting involved in long, complicated activities such as priority-setting. As a participant remarked, “*When you assign a person to a position, you should follow the hierarchical system. A new graduate cannot be the president of a university or college and have control over a full professor*” (P1). Some participants believed that some managers’ authority and will could be the main drive behind successful activities; therefore, putting them aside would automatically lead to the failure of a project. One of the participants declared, “*Today, setting up cohort studies is a priority for the Deputy of Research and Technology. Is it based on a scientific priority-setting activity or personal will? I think the latter is more important*” (P2).

According to the participants, so many researchers and managers do not have enough knowledge about the importance or methods of priority-setting. “*One of our problems is that we have a little knowledge about systematic priority-setting. Maybe we have done some priority-setting; but with what scientific method? Nothing. Experts must come together and evaluate different ways and propose a country-specific research method for priority-setting*,” insisted a participant (P2).

### Theme 2: Structural factors

In some participants’ opinion, “*one of limiting factors is centralised decision-making approaches*” (P4). Bureaucracy is another problem, as stated by a manager: “*Because of the top-down decision-making system, we cannot independently decide to do an intervention or change a process*” (P4). Establishing independent granting bodies was also recommended by some participants with the aim of enhancing the likelihood that priority-setting results could be implemented. A participant said, “*In our country, medical universities act simultaneously as a granting body, a research centre, and also a medical journal. It is corrupting and will waste financial resources. It is obvious that such an institution would allocate resources to projects that comply with wishes of faculty members, not problems of the community, and have greater likelihood of publication*” (P11).

As to whether stakeholders or the general public should be informed, almost all participants insisted on informing the first group. A participant argued, “*In my opinion, stakeholders, rather than the public, should be informed about results of priority-setting*” (P3). There were conflicting opinions about the role and efficacy of the media in disseminating scientific results. A participant strongly disagreed on using the media for this purpose: “*If you remember, when differentiated heart cells were produced for the first time at the Royan institute, it was reported in the TV news – showing a heart pounding. You would have said then, ‘Everyone needs a heart – go and get it right now!’*” (P1). Furthermore, the majority of participants maintained that, by observing some conditions, the media could play a constructive role in publicising research or priority-setting results. One of them declared, *“We don’t make use of the media appropriately. If we want to disseminate a scientific finding to the society, the media are the best way available –but as long as they are trained and briefed well*” (P5).

Nowadays, automated mechanisms are frequently used in the health system in Iran, for example, the Hospital Information System, the Electronic Health Record System (In Farsi “SEPAS”), the Integrated Income Information System (in Farsi “Sadjad”), and local online systems, in the evaluation of the performance of hospitals and Pajooheshyar (a system for registering all research projects in a university). Some interviewees pointed to the absence of these systems in research management. One of them said, “*There are a lot of research topics. If there was a software programme to codify these topics, you could easily monitor the compliance of proposed projects with priorities and prevent the duplication of efforts*” (P8).

Some participants argued that, in order to encourage innovation, market rule and demand-based research, it was necessary to establish a national innovation system and similar systems such as science and technology parks as well as incubators. An interviewee said, *“Government has an obligation to establish a national innovation system and its components such as science and technology parks, venture industries fund, and knowledge-based companies supporting laws*” (P7).

Stewardship was a factor that almost all of the interviewees regarded, in different expressions, as the research system’s ‘Achilles heel’. One of them declared that the research system should be reconstructed by research puzzles: “*In other countries, scientific departments have their own research puzzles and all resources and processes, such as priority-setting, are designed and provided based on solving problems*” (P17). Standardisation as a function of the research system was at the centre of some participants’ attention. *“We always highlight the applied research but never define it properly. When we don’t have any standard, we can’t govern anything. For example, there is no clear standard in the connection between medical universities and industries,*” commented a participant (P12). Clarification, along with transparency, was mentioned as another important function of stewardship. A participant asserted, “*What happened in Turkey as an improvement to health indices was not specifically related to the health sector. It partly happened in the economic sector in terms of transparency. Bad leadership was considered to be another reason for failure. Some years ago, CBPR* [Community Based Participatory Research] *was introduced in Iran and a meeting was held in the province of Ardabil. What happened to CBPR? We had a popular approach to it without any scientific dialog or collectivity. No body of knowledge was produced about it*” (P7).

Another subject that the participants emphasised was the availability of information and evidenced-based decision-making. One interviewee said, “*While a group of participants is involved in ranking items, another group should gather relevant information and knowledge gaps through reviewing literature*” (P7).

According to some interviewees, research in Iran cannot be demand based as long as universities, as centres of knowledge production, are isolated from industries. One of them said, “*No connection can I see between research and market. Our universities watch what is happening in great universities of the world and just simulates it; so does our market*” (P10).

Comparing the options selected by different types of participants in terms of decision level, it can be seen that 6 of the top 10 options are common to all three groups. Similar items are research priority-setting approaches, inappropriate leadership of research, using end-users as stakeholders, lack of National Innovation System, limiting research budgets to priorities and lack of evidence about research gaps. Except for the two cases that ranked 35th and 25th in the micro level in comparison with the rankings 7th and 10th at the macro and 13th and 10th at the meso level, the remaining two of the top 10 at macro were up to the 18th place at two other levels of decision-making hierarchy (Table 3).

**Table 3:** The themes proposed by the participants differ in the level of decision making and the ranking in each level

sub-themes	Macro	Meso	Micro	sub-themes	Macro	Meso	Micro
Priority-setting approaches	1	1	2	Rapid turnover of managers	19	25	16
Stewardship-Inappropriate leadership of research	2	2	1	Centralized decision making	20	33	31
Stakeholders: end users	3	8	3	Stewardship: No standardization	21	7	23
Inefficiency of faculties’ promotion criteria	4	6	12	Stakeholders: Policy makers	22	28	20
Lack of National Innovation System	5	3	4	Considering priority setting as a one-time activity	23	29	21
Limiting research budgets to priorities	6	5	9	Using an automated system	24	30	30
Lack of a efficacious evaluation system	7	13	35	Narrow time limit for priority setting	25	11	15
Scarcity of knowledge about health research priority-setting	8	15	18	Publicizing health research priority-setting results	26	20	19
Lack of evidences about research gaps	9	4	7	Stewardship-Lack of Transparency in other sectors	27	23	33
Stakeholders: ways of engagement	10	10	25	Stakeholders: NGOs	28	14	16
Individual willingness	11	9	17	Ignoring appealing mechanisms	29	26	22
Alignment with high level rule	12	12	24	Stewardship:Absence of a research map (research puzzles)	30	34	14
Generalization	13	22	8	Stakeholders: Funders	31	19	29
Commitment of managers	14	24	6	Meritocracy in management	32	32	13
Defining reliable PS criteria	15	18	5	Role of media	33	27	27
Defining scope of PS	16	21	25	Considering intellectual property	34	35	34
Scientific autonomy	17	15	11	Integration of heath with medical education	35	31	32
No relation with industries	18	17	27				

Numbers are ranks of proposed sub-themes by participants in different levels of decision making

### Theme 3: Motivational factors

This theme refers to viewpoints on supporting tasks of the national research system. An interviewee said, “*Some researchers are not interested in expressing their opinions because they are concerned about the lack of respect to intellectual property*” (P3). Faculty members’ promotion is based on published articles. A participant remarked, “*When we don’t define incentives properly, no university professor will be interested in priority-setting. He does research that would lead to ISI articles and more promotion scores, not priority-setting*” (P13).

Relating research and resources to each other was heard in most of the interviews’ words. In their opinion, it not only acts as a motivator factor but also as the final target of priority-setting. “*For instance, 70% of the research budget should be allocated to developmental and practical research*” (P8). Some researchers disagree on the allocation of all resources to priorities and assert that part of it should be preserved for innovation and scientific merit. For example, a participant declared, “*Our only way to do priority-setting activities is allocating budgets to priorities, but, some of them should be abandoned for innovation*” (P3).

### Theme 4: Process factors

Almost all of the interviewees emphasised the importance of setting criteria for priority-setting. A participant said, “*Criteria are necessary for priority-setting. The strength of COHRED lies in its criteria*” (P3).

The majority of the participants stated that priority-setting was a time-consuming task. One of them said, “*Typically, when superordinate organisations order subordinates to do priority-setting, they put pressure on them to do it as soon as possible. It could disrupt the process because the given time is generally irrational*” (P2). Another one believed that the time frame for performing priority-setting was interrelated to other aspects of the process: “*When you set priorities horizontally, you should consider a wide vision. For example, when we want to design epidemiological studies, we should have a 10-year time frame; but the time frame could be shorter in vertical prioritisation*” (P11). According an interviewee “*Priority-setting is not some once-and-for-all action. When you identify research priorities, some research will be conducted. So, you should specify if the research answered the research question. If positive, the given priority is omitted and another one substitutes for it. To do this process, priority-setting should be periodically revised*” (P3).

As was briefly mentioned above, generalisation is another characteristic of all priority-setting. Generalisation means presenting priority-setting results in terms of statements which encompass a large collection of items. One of the interviewees said, “*Suppose you selected ‘family physician’ as a priority. Practically, you didn’t say anything because almost one third of the health system could be placed in the domain of ‘family physician’*” (P18).

The composition of stakeholders and their involvement methods was another sub-theme. “*You cannot expect that all stakeholders hold the highest ranks. For example, second and third rank representatives don’t have decision-making authority. Here, we must remember that participation doesn’t mean physical presence. Sometimes continuous notification about discussions or decisions is more appropriate*” (P21). Involving end-users and community members was one of the most conflicting subjects among the interviewees. On the one hand, there were participants who insistently regarded the community as the main stakeholder. One participant declared, “*In my opinion, certainly, we should involve people, especially end-users, in priority-setting. Although they are often wrong, they are those who direct us*.” On the other hand, some participants confined the involvement of the community to the needs-assessment phase of priority-setting. “*Opinions of the community should be extracted by doing surveys or completing questionnaires in the need-assessment phase*” (P22).

Some interviewees had the idea that non-governmental organisations (NGOs) were better players in participatory research. One of them had the idea that “*we should identify public brokers to act as an intermediary between researchers and laymen in community-based participatory research. These brokers include members of City or Village Islamic Councils in Iran or NGOs*” (P8). Nevertheless, there were lots of participants who disagreed on the constructive role of NGOs in the Iranian context: “*Our NGOs are not completely detached from governmental budgets and constraints. They are still worrying about leaving a comment that could lead to their exclusion from state resources or certificates*” (P4).

On the one hand, almost all of the participants were unanimous about the role of funders in priority-setting. “*If our PhD dissertations were formed on the basis of financial grants as everywhere else* [in the world]*, this would automatically direct our research to priorities. That is because funders usually allocate their financial grants to their priorities*” (P17). As usual, there were some concerns about funders’ intentions. One interviewee said, “*Funders usually have a financial relationship with researchers or health providers with the intention of achieving desirable results.*”

Some participants stated that scientific autonomy was one of the most serious flaws of researcher-based priority-setting. According to a participant, “*culturally, faculty members are used to insisting on their own words* [uncompromisingly]. *Their interests are more important than everything else. Their insistence is irrational*” (P15). The absence of research lines was another problem which was mentioned by some participants. A participant said, “*I know researchers who submit research proposals in any scientific field, from spaceship to hair. They don’t have any research line*” (P17).

Although policy-makers have an important role in all stages of priority-setting, according to some participants, their role could be counter-productive in general. A participant argued, “*Policy-makers usually decide on the basis of political intentions. So, they don’t care about your hard-earned evidence*” (P22).

Establishing an appeal mechanism was another subtheme, which means producing opportunities so that priority-setting results could be criticised and improve through feedback. An interviewee said, “*It improves priority-setting; but it should be done by those who are not directly involved in research – I mean a third party, for example*” (P14).

Some interviewees believed that priority-setting should be in line with superordinate documents. This acts like a lantern, which shines and leads outcomes of the prioritised research to organisational goals. “*Now we have different national documents. These documents show what are highlighted at the national level and how I should prioritise my institutional activities*” (P2).

Monitoring and evaluation are effective in directing attention to priority-setting in research in an institution. These are carried out by completing the priority-setting process and conducting the prioritised research. An interviewee said, “*No monitoring and evaluation mechanism has been developed for our priority-setting activities. What percentage of priorities is properly defined?*” (P6).

There are different priority-setting methods, which could be divided into two main groups, namely consensus based and metrics based. Although a large number of the interviewees had the idea that pure consensus-based methods were not great and were just acceptable in information-restricted situations, some indicated that almost all shortcomings were related to methodology. “*If our consensus-based method, such as a discussion meeting or expert panel, had acceptable credibility and accuracy, its results could be reliable. In my opinion, consensus-based methods are not inherently weak*” (P15). If we regard consensus-based approaches as the main approaches to participation, we will encounter the common problem of the dominance of influential members. A participant declared, “*When a powerful person is among stakeholders, others definitely won’t express their real opinions. They will just concur with him but, when they leave the session, nothing will change. They still hold their own opinions*” (P14). To solve the latter problem, the participants recommended solutions. For example, one of them said, “[We should] *choose stakeholders of the same level rather than passive ones. An in-depth interview is a proper method for solving this problem*” (P5). Another problem that could be important in consensus-based approaches is researcher skills. “*Researchers don’t learn how to communicate with laymen or community members. We learn a lot of things about research methodology but receive no training in public relations*” (P5).

## Discussion

Herein, we interviewed 23 participants experienced in priority-setting in health research in order to draw up an exhaustive list of factors hindering and facilitating priority-setting in health research in Iran. The most important factors were the composition of stakeholders and the range as well as quality of their involvement; provision of definitions of criteria for research priority-setting; consideration of the fairness and legitimacy of decision-making; selection of an efficient method for research priority-setting; consideration of superordinate rules; and weaknesses in stewardship.

There are a large number of studies on the prioritisation of health research topics and the way it should be performed in Iran; however, based on research conducted by our team, this is the only study in Iran evaluating the knowledge and experiences of individuals who deal with this process one way or another. Obviously, foreign scientific literature has a lot to tell us about research prioritisation. Nevertheless, since priority-setting is context based, we hope the current study, which is unique in social, cultural and political terms, will not only contribute to the prioritisation process at Iran but also broaden experiences of other countries with similar conditions with respect to the prioritisation of health research topics.

A myriad of strategies should be considered to make research priority-settings more efficient and acceptable. Leadership that is not limited only to the process of priority-setting is an important factor. Moreover, some aspects of leadership, such as meritocracy, management stability or ranking managers’ commitment, cannot be achieved easily and some intermediate measures should be taken, including binding legislation, providing inclusion training in priority-setting, shifting promotion indicators from the exclusive dependence on publishing articles in high impact factor journals to the provision of solutions for health problems, giving policy-makers policy briefs, and conducting commissioning research.

The prioritisation of research topics is a process that could be performed at international, national and regional levels. Furthermore, this process might be limited to one aspect, such as prevention, or include all aspects ranging from prevention to treatment and rehabilitation. In addition, the prioritisation of research topics could be discussed with regard to disease, risk factors or health issues. The research priority-setting could comprise either particular types of research, such as basic research, or all types of research on disease, including clinical, sociocultural and health systems research. Determining the scope and level of research priority-setting is an important phase that should not be undervalued, otherwise it would affect the way different prioritisation stages, such as the composition of stakeholders, the budget, and necessary information, are designed.

An important finding was related to participants’ opinions about the composition of stakeholders and methods of their involvement in research priority-setting in Iran. Consistent with our participants, who mentioned different groups of stakeholders, other studies also recommend involving, at least four main groups, namely researchers, policy-makers and decision-makers, healthcare providers, and community members [30–32]. Due to some discrepancies between clinical researcher intentions and patient needs, some researchers believe that end-users of health services in Iran must be regarded as stakeholders [33–36]. Somewhat similar to our participants, some researchers have the idea that a more developmental approach should be adopted to put the community on an equal footing with university researchers. Hence, it is recommended that the participation of the community in research priority-setting be limited to the problem finding phase of priority-setting [37]. Another group of beneficiaries are politicians, who are usually overlooked when priority-setting is performed in developing countries. The role of politicians is significant since not only could they generate relevant ideas but they could also increase the likelihood of implementing them [38]. Funders have the heavy responsibility to make research more applicable by, for example, providing information about how they decide to support research, ensuring that collaboration will reduce expenditures and that the research does not duplicate existing evidence, and justifying research on existing evidence [39]. Like our participants, other studies also recommend involving funders in processes of priority-setting in research so as to achieve these goals [30]. Dissociation of industry from research has been introduced as an obstacle to target-based planning and evidence-based decision-making processes such as research priority-setting in Iran [28]. Consistent with our participants’ viewpoints, various studies regard relevant industries as important beneficiaries of research priority-setting [7, 31, 32]. To conclude, the participation of a broad range of stakeholders is a critical issue. It is recommended that, based on the scope and level of priority-setting, different forms of participatory methods, including Delphi, interview and focus group discussions, be taken into account to deal with potential problems, including the dominance of influential members, profit-based intentions of funders or industries, researchers’ scientific aggrandisement, and involuntary participation of representatives of a community.

The participants made the point that criteria should be used to rank recommended priorities, which was consistent with previous studies [30, 33]. Using criteria could organise the reasoning process that makes priority-setting in research a challenging phenomenon, and could prove useful, particularly in low-information settings such as Iran. Furthermore, using criteria makes priority-setting clear (to stakeholders), more measurable and transparent [34], and in line with standards. Criteria must be rooted in stakeholders’ values, existing resources and institutional strategic directions and goals [35]. Researchers recommend that the determination of criteria be followed as a weighting process to include the abovementioned items [36]. To be adjustable to all contexts and more applicable, criteria should be set in a flexible process [36].

A further point made by some researchers regarded the lack of respect to intellectual property, which makes stakeholders have superficial involvement in the research priority-setting process [40]. Since priority-setting is a dynamic process and ideas should be almost always drawn from participants, an unrestricted environment ought to be provided. To this end, involving those who have ideas in conducting research, implementing a monitoring system, issuing a letter of encouragement, and providing feedback [41, 42] through publishing newsletters or issuing bulletins [26] are strategies that have been recommended.

Fairness and legitimacy, introduced by Daniels and Sabin as the theory of “*accountability for reasonableness*” [43], are crucial features of decision-making. Four components of this theory are transparency/publicity, relevance, revision and enforcement. Transparency has been mentioned in several priority-setting experiences as a necessary component that could induce ownership among all stakeholders [5, 44]. The absence of systematic appeal or feedback mechanisms has been observed in innumerable activities in Iran [45]. In summary, transparency and revision are the most important elements of research priority-setting; despite being often overlooked, these could be attained through clarity of methods, dissemination of results and revisions.

Methods for priority-setting in research range from pure consensus-based to pure metric approaches. The pure consensus-based method, also called an implicit or interpretive approach, completely relies on participants’ viewpoints, in which some consensus-building tools and techniques, including Delphi and nominal group and focused group discussions, could be used. These approaches, which are commonly used for performing priority-setting in research in large governmental agencies [46], neither lead to the best application of resources nor are found ethically acceptable [47]. Pure metric approaches use only data such as Disability Adjusted Life Year or econometric data for prioritisation. Although they depend on definitive and strong evidence, pure metrics omit stakeholder judgments [46]. Based on numerous studies, as well as the present study, mixed approaches involving informed judgments made on the basis of information are better options in settings, such as Iran, where evidence is not available [46].

Considering superordinate laws, including national development laws or sectoral laws on health, institutional goals and strategies were recommended by our participants and others [48, 49]. Some researchers believe that performing research priority-setting on the basis of a strategic plan could eliminate the hardness of high level decision-makers’ participation. Some consistent opinions also express that strategic research priorities should be identified based on long-term perspectives [29, 50, 51]. Other proponents believe that awareness of policies, plans, previous efforts and activities of institutions helps to identify needs in a more rational and wider framework [52]. Overall, considering superordinate documents could be useful in providing policies and legislative structures, directing priorities and creating mechanisms to support research [8, 53]. Further, research priority-setting decisions should be based on reasons that are grounded in clear values and stakeholders should gain insight not only about goals and rationales of priority-setting, but also about missions, visions, values and strategic plans of organisations [5, 7, 54]. Other studies mentioned that the lack of accountability to high-level goals and strategic directions could cause imbalances in research investment [36, 55]. In conclusion, although there are some technical and methodological shortcomings in the preparation of strategic plans, especially in developing countries [56, 57], they cannot justify ignoring these valuable guidelines.

The rapid turnover of managers is another major barrier to developing research capacity, especially in Iran [7, 26, 58–60]. In addition to a rapid turnover, health-research managers suffer from a degree of unawareness of the philosophy and methods of priority-setting in research. Academic incentives have been confined to the publication of articles in high impact factor journals without any attention to their alignment with priorities or problems of the community [61]. In order to apply evidence-based decision-making, priority-setting requires reliable data; however, as mentioned above, priority-setting in Iran is mostly affected by the lack of data on magnitude, burden, costs and accessibility, among other factors. This automatically directs research priority-setting toward pure consensus-based approaches, which, due to its subjectivity, is not attractive to stakeholders and is easily criticised by them.

The community and larger groups of stakeholders deserve to be aware of the logic behind decisions on priorities. This could be achieved by mutual cooperation through guided media, social networks, bulletins or newsletters. Received feedback creates excellent foundations for revising research priority-setting results. Naturally, in order to achieve this, the entire process of research priority-setting should be documented in detail.

An important phase of all research priority-setting activities usually ignored in Iran is that of monitoring and evaluation. Indeed, there are mechanisms that monitor the accuracy of process elements, including selecting stakeholders, gathering relevant evidence and avoiding generalisations. However, it is necessary to establish an office to monitor the allocation of a major share of resources to priorities, alignment of advanced research with priorities, an increase in research collaboration, and an increase in the provision of policy briefs for policy-makers and guidelines for healthcare providers.

Although resource scarcity is one of the most important problems in the country, attention to research priority-setting is not considered as a route to prevent spoilage of resources. Repetitive studies not only cause loss of financial resources, but also eliminate other important resources such as time. Beside repetition, the dissociation of research from a strategic and targeted plan and the lack of definition of research goals in medical universities have not encouraged these institutions to concentrate activities on a particular area of health problems. The inadequate stewardship role of government in assigning special research tasks to institutions in the form of general puzzles is another cause of waste, as is the separation between researchers and policy-makers. Politicians do not consider themselves as required to use the knowledge of researchers to solve health problems, and researchers, on the other hand, are reluctant to engage themselves with the world of politics. There are no mandatory laws enforcing industries to use the knowledge and expertise of researchers who are active in medical science universities.

## Conclusions

The results of the present study suggest that research priorities in Iran are well understood and identified at all levels of decision-making, and it is now time to put in place practical support to resolve the recognised issues. Despite restating what is known as the factors affecting priority-setting in international documents, our interviewees declared some new and country-specific factors, including a lack of senior management commitment to priority-setting, dissociation between industry and research, and non-stewarded mechanisms for priority-setting such as institutional legislation, centralised and top-down decision-making processes, and time limitations. Due to its complexity, more work needs to be performed to produce a greater body of knowledge regarding the structural constraints affecting priority-setting in Iran. Furthermore, impact analysis studies on previous priority-setting efforts in Iran could inform decision-makers about the reasons leading to their side-lining.

## Abbreviations

COHRED: Council on Health Research for Development
NGO: non-governmental organisation
